# Mortalin (GRP75/HSPA9) Promotes Survival and Proliferation of Thyroid Carcinoma Cells

**DOI:** 10.3390/ijms20092069

**Published:** 2019-04-26

**Authors:** Dmytro Starenki, Nadiya Sosonkina, Seung-Keun Hong, Ricardo V. Lloyd, Jong-In Park

**Affiliations:** 1 Department of Biochemistry, Medical College of Wisconsin, Milwaukee, WI 53226, USA; dstarenki@hudsonalpha.org (D.S.); nsosonkina@hudsonalpha.org (N.S.); skhong@mcw.edu (S.-K.H.); 2 Department of Pathology and Laboratory Medicine, University of Wisconsin, Madison, WI 53792, USA; rvlloyd@wisc.edu

**Keywords:** mortalin, HSPA9, mitochondria, Mito-CP, MTC, PTC, FTC, ATC

## Abstract

We previously reported that upregulation of mortalin (HSPA9/GRP75), the mitochondrial HSP70 chaperone, facilitates tumor cell proliferation and survival in human medullary thyroid carcinoma (MTC), proposing mortalin as a novel therapeutic target for MTC. In this report, we show that mortalin is also upregulated in other thyroid tumor types, including papillary thyroid carcinoma (PTC), follicular thyroid carcinoma (FTC), and anaplastic thyroid carcinoma (ATC), and that mortalin depletion can effectively induce growth arrest and cell death in human PTC (TPC-1), FTC (FTC133), and ATC (8505C and C643) cells in culture. Intriguingly, mortalin depletion induced varied effects on cell cycle arrest (G0/G1 phase arrest in TPC-1 and C643, G2/M phase arrest in 8505C, and mild G2/M phase arrest with increased sub-G0/G1 population in FTC133) and on the levels of TP53, E2F-1, p21^CIP1^, p27^KIP1^, and poly (ADP-ribose) polymerase cleavage in these cells, suggesting that thyroid tumor cells respond to mortalin depletion in a cell type-specific manner. In these cells, we also determined the efficacy of triphenyl-phosphonium-carboxy-proxyl (Mito-CP) because this mitochondria-targeted metabolism interfering agent exhibited similar tumor suppressive effects as mortalin depletion in MTC cells. Indeed, Mito-CP also induced robust caspase-dependent apoptosis in PTC and ATC cell lines in vitro, exhibiting IC_50_ lower than PLX4032 in 8505C cells and IC_50_ lower than vandetanib and cabozantinib in TPC-1 cells. Intriguingly, Mito-CP-induced cell death was partially rescued by mortalin overexpression, suggesting that Mito-CP may inactivate a mechanism that requires mortalin function. These findings support the significance of mortalin and mitochondrial activity in a broad spectrum of thyroid cancer.

## 1. Introduction

Thyroid cancer, the most common neoplasm of the endocrine system, is the seventh most frequent human malignancy and its incidence is increasing more rapidly than any other cancers. Thyroid cancer is mainly treated by surgery and radioiodine remnant ablation but these therapies are effective only for non-metastasized primary tumors. Most of metastatic or relapsed thyroid tumors are incurable, demanding advanced therapeutic modalities for patient survival (reviewed in [1,2]). 

Depending upon the cell of origin and histological characteristics, thyroid carcinomas are generally classified as papillary thyroid carcinoma (PTC), follicular thyroid carcinoma (FTC), anaplastic thyroid carcinoma (ATC, undifferentiated), and medullary thyroid cancer (MTC). PTC, FTC, and ATC arise from the follicular thyrocytes whereas MTC is the only parafollicular C-cell-derived tumor, constituting the minor fraction of thyroid malignancies [1,2]. Various molecular alterations drive thyroid carcinogenesis. For example, about 65% to 70% of the follicular thyrocyte-derived tumors exhibit genetic or epigenetic alterations in the Ras/Raf/MEK/mitogen-activated protein kinase (MAPK) pathway, e.g., mutations in *BRAF* and *NRAS*, and the phosphatase and tensin homolog/phosphatidylinositol three-kinase/AKT pathway, while about 20% of PTC cases exhibit chromosomal rearrangements of the kinase domain of rearranged during transfection (RET/PTC) (reviewed in [3]). Meanwhile, constitutively active *RET* mutations drive about 95% of hereditary MTC and about 50% of sporadic MTC cases [4,5]. Although the plethora of molecular information has allowed the design of advanced therapeutic strategies for thyroid cancer, significant limits still remain in current strategies for targeted therapy and additional therapeutic targets are required. 

Mortalin (HSPA9/GRP75/PBP74) is a member of the heat shock protein (HSP) 70 family which also includes the cytosolic heat shock cognate 71 kDa (HSC70/HSPA8) and the endoplasmic reticulum chaperone, BiP/HSPA5 [6]. Although originally identified as a mitochondrial molecular chaperone [7], mortalin is also detected in different subcellular compartments, suggesting its diverse functions in cells [8,9,10]. Mortalin is often overexpressed in cancers, including the tumors of colon, liver, brain, breast, and skin, and growing evidence suggests that mortalin is an important regulator of tumor cell growth and survival [9,10,11,12]. We have recently reported that mortalin is upregulated in human MTC and that RNA interference or inhibition of mortalin can effectively suppress the human MTC cell lines in culture as well as in mouse xenografts [13,14]. Intriguingly, depletion of mortalin induced not only growth arrest but also robust cell death by disrupting mitochondrial bioenergetics and redox balances, suggesting its important role in mitochondria for MTC cell survival [13,14]. Subsequently, we discovered that the mitochondria-targeted metabolic interfering agent, triphenyl-phosphonium-carboxy-proxyl (Mito-CP), can also effectively suppress MTC cells via similar mechanisms as induced by mortalin targeting [15,16]. These findings from MTC led us to evaluate the significance of mortalin and the potency of Mito-CP in other thyroid tumor types. 

In this study, we demonstrate that mortalin is also upregulated in human PTC, FTC, and ATC tissues and subsequently evaluate its importance in a subset of human PTC and ATC cell lines that harbor RET/PTC or B-Raf^V600E^. Our data demonstrate that mortalin is necessary for proliferation and survival of these tumor cells and that Mito-CP effectively suppresses these cells with IC_50_ higher than FDA-approved kinase inhibitors, PLX4032, vandetanib, or cabozantinib. As such, our findings suggest expanded significance of mortalin and mitochondria targeting in different thyroid tumor types.

## 2. Results

### 2.1. Mortalin Levels are Upregulated in PTC, FTC and ATC Patient Tissue Biopsies

**Figure 1:**
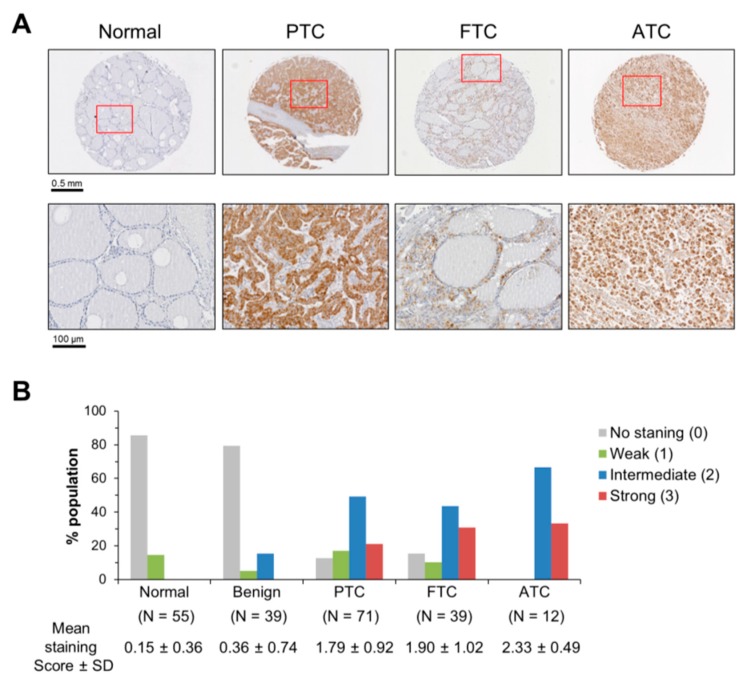
Mortalin is upregulated in human thyroid cancer. (**A**) Representative immunohistochemical analysis images of mortalin protein in normal thyroid, papillary thyroid carcinoma (PTC), follicular thyroid carcinoma (FTC), and anaplastic thyroid carcinoma (ATC). (**B**) Scores of mortalin expression in patient tissue biopsy specimens. *p* > 0.05 for benign tumor vs. normal; *p* < 0.001 for PTC vs. normal; *p* < 0.001 for FTC vs. normal; *p* < 0.001 for ATC vs. normal, Kruskal–Wallis test (nonparametric ANOVA) with Dunn post-test for multiple comparison.

To examine mortalin levels in thyroid cancer, we conducted immunohistochemical analysis of 71 cases of PTC, 39 cases of FTC, 12 cases of ATC, 39 cases of benign thyroid tumor patient tissues in comparison with 55 normal thyroid tissues. Using a mortalin-specific antibody validated for IHC in our previous reports [12,13], we found that mortalin protein levels were significantly upregulated in PTC, FTC, and ATC but not in the benign tumor tissues (Figure 1A,B). This expression was relatively high when compared to the levels of mortalin in MTC tissue specimens (mean staining score 1.08 ± 0.16, *p* < 0.0001, Mann–Whitney test) that we reported previously [13]. These data suggested extended significance of mortalin in different thyroid tumor types, leading us to investigate the role of mortalin in different thyroid tumor cell lines.

### 2.2. Mortalin Depletion Induces G0/G1 Phase-Cell Cycle Arrest and TP53 Upregulation in the PTC Cell Line, TPC-1

**Figure 2:**
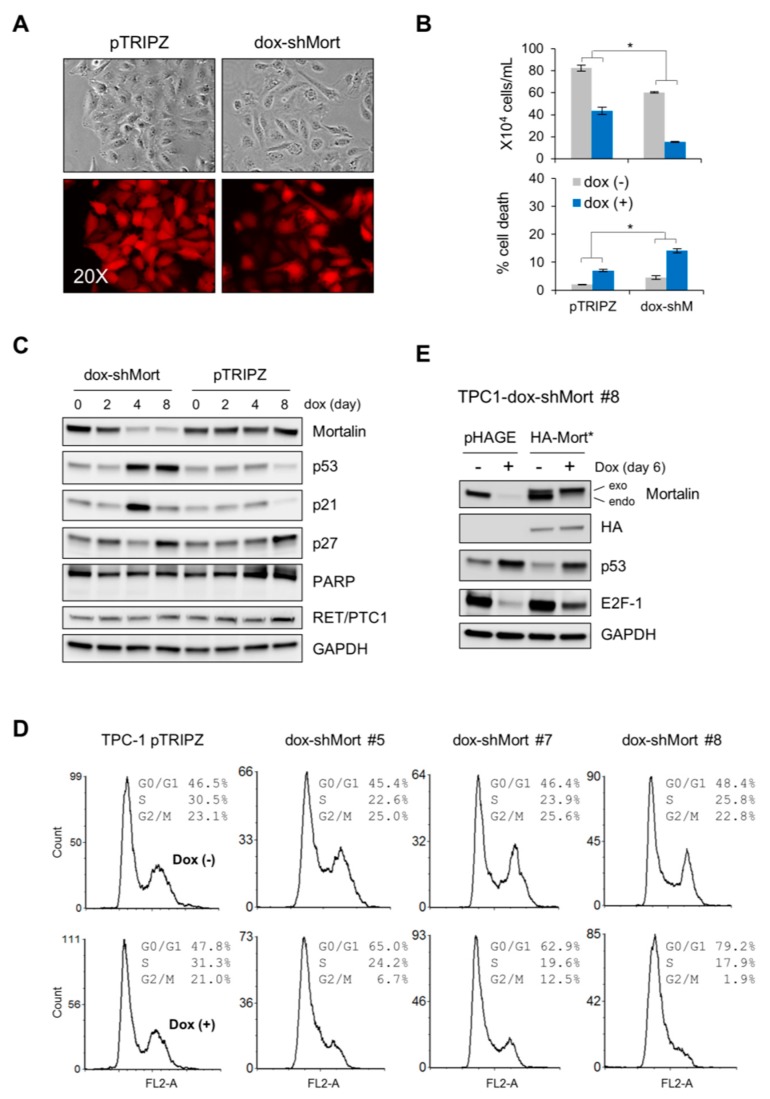
Mortalin knockdown induces growth inhibition in the PTC cell line, human PTC (TPC-1). (**A**–**C**) PTC-1 cells were infected with the pTRIPZ lentiviral vector harboring a doxycycline (dox)-inducible shRNA construct that targets mortalin mRNA (dox-shMort), or with the empty pTRIPZ virus. (**A**) Dox-induced red fluorescent protein (RFP) expression indicates infection efficacy. (**B**) Trypan blue exclusion analysis of live and dead cells at dox treatment day 8 (mean ± SD, *n* = 3), * *p* < 0.05. (**C**) Western blot analyses of total cell lysates harvested after dox treatment for indicate time. Glyceraldehyde-3-phosphate dehydrogenase (GAPDH) was used as the control for equal amounts of protein loading. (**D**) Cell cycle analysis of different clones of TPC-1 stably infected with pTRIPZ-dox-shMort (#5, #7, and #8) after 8 day dox treatment. (**E**) Cells of TPC-1 dox-shMort clone #8 were infected with lentiviral pHAGE expressing non-shMort-targetable mortalin mRNA (Mort*). Western blotting of total cell lysates indicates that HA-Mort* can abrogate mortalin knockdown effects on p53 and E2F1. Empty pHAGE was used as the control.

Using a lentiviral doxycycline-inducible small hairpin RNA (shRNA) expression system (dox-shMortmir) that target mortalin mRNA, the RET/PTC-expressing TPC-1 cells were transduced for inducible mortalin depletion. As determined by morphology and the trypan blue exclusion analysis, doxycycline treatment reduced the viability of dox-shMortmir-infected cells more significantly than that of control virus-infected cells (Figure 2A,B). Along with these effects, our time-course Western blotting revealed that mortalin depletion led to substantial increases in TP53 and p21^CIP1^ (a cyclin-dependent kinase inhibitor transcriptionally regulated by TP53) levels in TPC-1 cells, although this p21^CIP1^ upregulation was not sustained along the time-course (Figure 2C). However, mortalin depletion did not affect the levels of p27^KIP1^ (Figure 2C; p16^INK4a^ was undetectable). Under these conditions, the cleavage of poly (ADP-ribose) polymerase (PARP), a caspase-dependent apoptosis marker [17], did not occur while RET-PTC1 levels were not affected (Figure 2C).

Next, we derived multiple clones of TPC-1 stably harboring dox-shMort by puromycin selection and determined which of the mortalin knockdown-induced molecular markers were segregated with viability loss. In multiple progenies, mortalin depletion consistently increased G0/G1-phase population while decreasing the populations in S- and G2/M-phases (Figure 2D; knockdown efficiency shown in Figure S1). Consistent with their parental cells, these clones exhibited TP53 and p21^CIP1^ upregulation in response to mortalin depletion, along with varied effects on p27^KIP1^ expression and PARP cleavage (Figure S1). Moreover, as determined with one of these TPC-1-dox-shMort clones, exogenous expression of a mortalin gene engineered to avoid dox-shMort (HA-Mort*) partially abolished dox-shMort-induced TP53 upregulation and downregulation of the S-phase transcription factor E2F1 (Figure 2E), supporting the requirement of mortalin for TPC-1 cell viability.

### 2.3. Mortalin Depletion Suppresses the ATC Cell Lines, C643 and 8505C, and the FTC Cell Line, FTC133, via Cell Cycle Arrest in Different Phases

**Figure 3:**
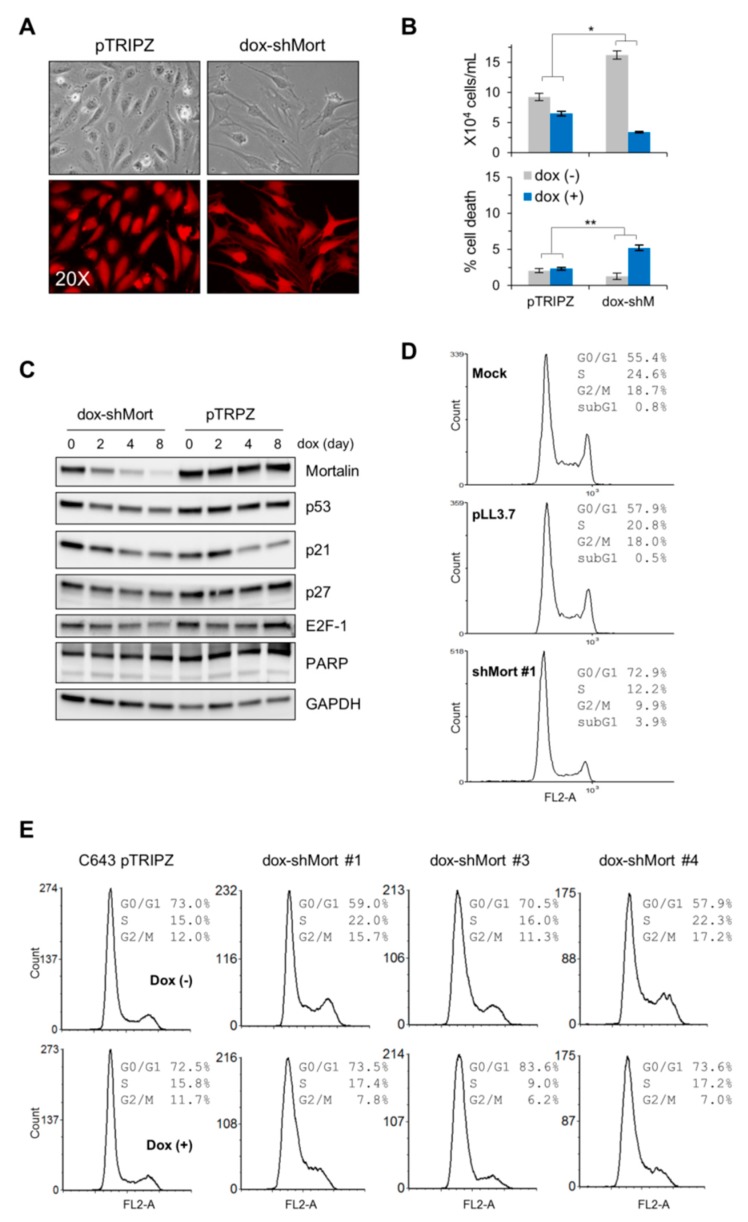
Mortalin knockdown induces growth inhibition in the ATC cell line, C643. (**A**–**C**) C643 cells were infected with the lentiviral pTRIPZ-dox-shMort or the empty pTRIPZ. (**A**) Dox-induced RFP expression indicates infection efficacy. (**B**) Trypan blue exclusion analysis of live and dead cells at dox treatment day 8 (mean ± SD, *n* = 3), * *p* < 0.05; ** *p* < 0.005. (**C**) Western blot analyses of total cell lysates harvested at indicated dox treatment days. GAPDH is the control for equal amounts of protein loading. (**D**) Cell cycle analysis of C643 cells infected for 4 days with the lentiviral pLL3.7 expressing shRNA that targets mortalin mRNA (shMort #1). shMort#1 and pTRIPZ-dox-shMort target different regions of mortalin mRNA. Uninfected (mock)- or empty virus-infected cells were used for comparison. Knockdown efficiency is shown in Supplemental Figure S2. (**E**) Cell cycle analysis of different clones of C643 stably infected with pTRIPZ-dox-shMort (#1, #3, and #4) after 8 day dox treatment. Western blot data of these clones are shown in Supplemental Figure S3.

Next, we determined the effects of mortalin depletion in the B-Raf^V600E^-expressing ATC lines, C643 and 8505C, and the PTEN-deficient (R130*) FTC line, FTC133. As determined by the trypan blue exclusion analysis, dox-shMort-mediated mortalin knockdown significantly suppressed the viability of C643 cells in culture (infection efficiency, viability, and knockdown efficiency shown in Figure 3A–C, respectively). Consistent with this, transient shRNA expression system (shMort #1) that target a different region of mortalin mRNA substantially increased G0/G1-phase population, along with mildly increased sub-G0/G1 phase population, while decreasing S- and G2/M-phase populations (Figure 3D; knockdown efficiency shown in Figure S2). 

Although this effect of mortalin depletion on cell cycle in C643 cells was similar as observed in PTC-1 cells, our time-course Western blot analysis revealed that mortalin depletion affects TP53 and other cell cycle regulators quite differently in C643 cells. Mortalin depletion did not upregulate the levels of TP53, p21^CIP1^, or p27^KIP1^ in C643 cells, which was contrasted with dox-shMort effects in TPC-1 cells (Figure 3C). Under these conditions, E2F1 was only mildly downregulated while the cleavage of PARP did not occur and p16^INK4a^ was undetectable (Figure 3C). When clonally selected C643 dox-shMort progenies were analyzed, all of these clones consistently exhibited substantially increased G0/G1 and decreased S-phase and G2/M-phase populations in response to mortalin depletion (Figure 2D; knockdown efficiency shown in Figure S3). Moreover, consistent with unselected total population, these clones did not upregulate TP53 and p21^CIP1^ in response to mortalin depletion. However, although varied, p27^KIP1^ upregulation and PARP cleavage were observed in these clones (Figure S3).

**Figure 4:**
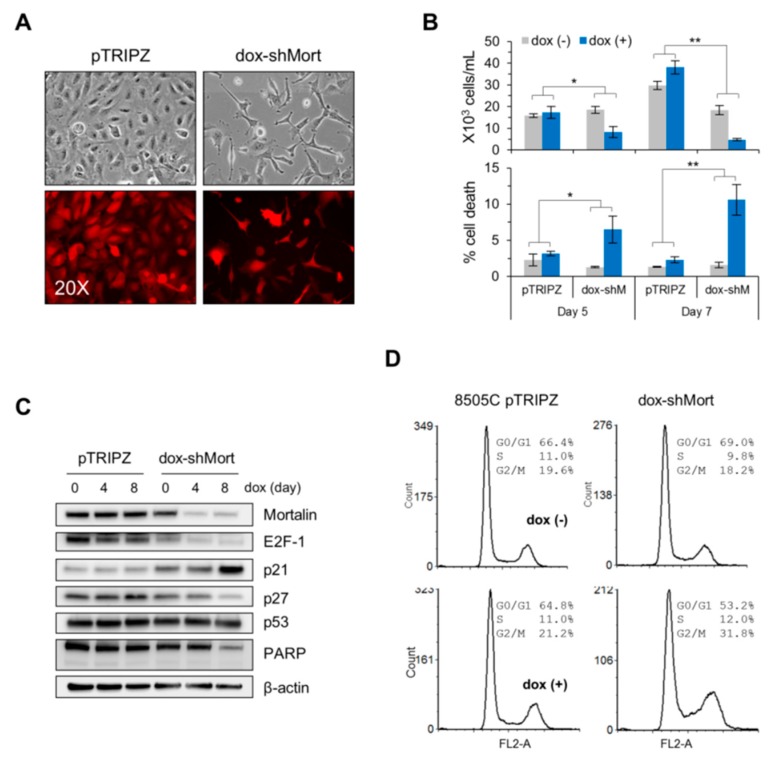
Mortalin knockdown induces growth inhibition in the ATC cell line, 8505C. (**A**–**D**) 8505C cells were infected with the lentiviral pTRIPZ-dox-shMort or the empty pTRIPZ. (**A**) Dox-induced RFP expression indicates infection efficacy. (**B**) Trypan blue exclusion analysis of live and dead cells at dox treatment day 5 and 7 (mean ± SD, *n* = 3), * *p* < 0.05; ** *p* < 0.005. (**C**) Western blot analyses of total cell lysates harvested at indicated dox treatment days. β-actin is the control for equal amounts of protein loading. (**D**) Cell cycle analysis after 8 day dox treatment.

Contrary to C643 cells, the ATC cell line 8505C exhibited different cell cycle profiles in response to mortalin depletion, although these cells exhibited consistent effects on viability loss (Figure 4B; knockdown efficiency shown in Figure 4C). In 8505C cells, mortalin depletion did not increase but decreased G0/G1 populations while substantially increasing G2/M-phase population and, to a lesser extent, S-phase population (Figure 4D). Moreover, contrary to C643 cells, mortalin depletion in 8505C cells induced significant p27^KIP1^ upregulation and downregulation of E2F1 and total PARP proteins while not affecting the levels of TP53 and p21^CIP1^ (Figure 4C). p16^INK4a^ was undetectable under this condition. 

**Figure 5:**
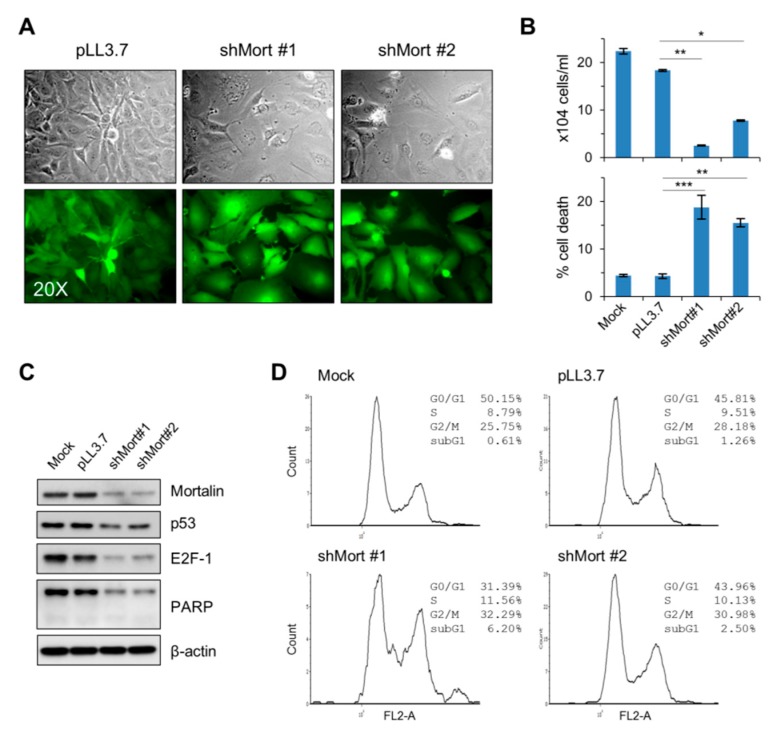
Mortalin knockdown induces growth inhibition in the FTC cell line, FTC133. (**A**–**D**) FTC133 cells were infected with pLL3.7 lentivirus expressing two different shRNAs that target mortalin mRNA (shMort #1 and shMort#2). (**A**) Green fluorescent protein expression indicates infection efficacy. (**B**) Trypan blue exclusion analysis of live and dead cells at infection day 4 (mean ± SD, *n* = 3), * *p* < 0.05; ** *p* < 0.005. (**C**) Western blot analyses of total cell lysates harvested at day 4. β-actin is the control for equal amounts of protein loading. (**D**) Cell cycle analysis at infection day 4.

In FTC133 cells, transient mortalin knockdown using two different shRNA system (shMort #1 and shMort #2) consistently suppressed their viability in culture (infection efficiency, viability, and knockdown efficiency shown in Figure 5A–C, respectively). In this cell line, mortalin depletion decreased G0/G1-phase populations while mildly increasing sub-G0/G1-, S-, and G2/M-phase populations (Figure 5D). Along with these effects, similarly as in 8505C cells, mortalin depletion in FTC133 induced substantial downregulation of E2F1 and total PARP levels while mildly downregulating TP53 levels (Figure 5C; p16^INK4a^, p21^CIP1^, and p27^KIP1^ were undetectable). These data demonstrate that mortalin depletion can effectively suppresses the viability of PTC, ATC, and FTC tumor lines in culture, possibly via a mechanism selected in a cell type-specific manner.

### 2.4. Mitochondria-Targeted Agent, Mito-CP, can Effectively Suppress Survival of PTC-1, C643, and 8505C Cells In Vitro

**Figure 6:**
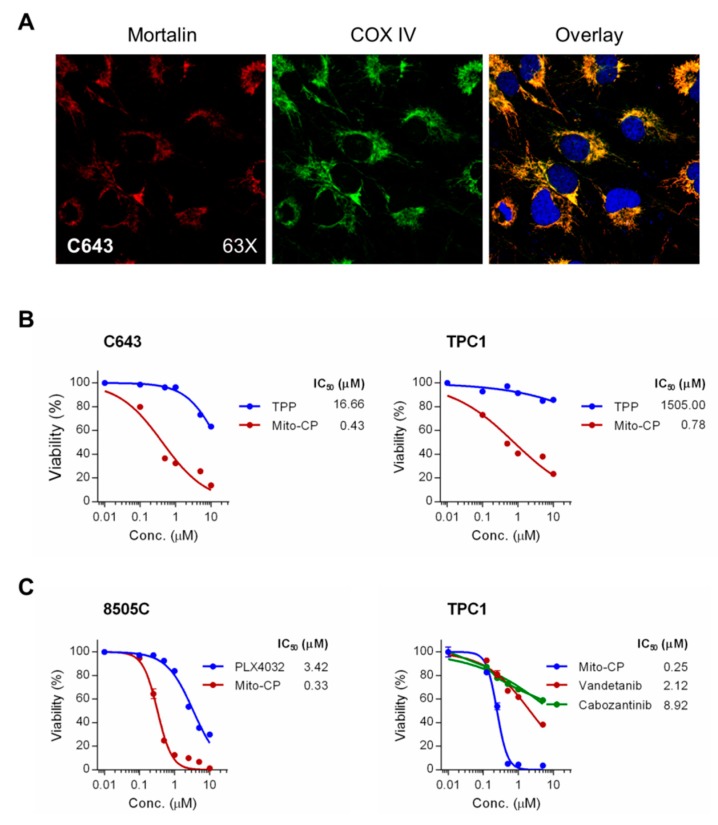
Mito-CP suppresses viability of PTC and ATC cells. (**A**) Immunofluoresence analysis of mortalin localization in C643 cells. cytochrome *c* oxidase (COX IV) serves as a marker specific to mitochondria. Areas of overlap are seen as yellow. (**B**) C643 and TPC-1 cells in 96-well plates were treated with serially increasing doses of Mito-CP or the control compounds, TPP, for 48 h. Cells were then allowed to recover in drug-free fresh medium for 24 h prior to measuring cell viability by 3-(4,5-dimethyl-2-thiazolyl)-2,5-diphenyltetrazolium bromide (MTT) assay. Data (mean ± SD, *n* = 4) are expressed as the percentage of untreated control. (**C**) Cells of 8505C and TPC-1 were treated with increasing doses of Mito-CP, PLX4032, vandetanib, or cabozantinib for 48 h. Cells were then allowed to recover in drug-free fresh medium for 24 h prior to MTT assay.

We previously showed that (i) mortalin is mainly localized in mitochondria in MTC cells [13]; (ii) that mitochondrial damage was a key mechanism by which mortalin depletion suppressed MTC cells [13]; and (iii) that interfering with mitochondrial redox and bioenergetics using the mitochondria-targeted agent, Mito-CP, was as effective as mortalin depletion in suppressing MTC cells [14,15,16]. Given the current data demonstrating the effective growth inhibitory effects of mortalin depletion in non-MTC thyroid cancer cell lines above, we determined whether Mito-CP would also effectively suppress these cells. This question was further rationalized by highly overlapping signals of mortalin and the mitochondrial marker, cytochrome *c* oxidase (COX IV) in C643 cells (overlap coefficient = 0.94), which suggested an important role of mortalin in mitochondria in these cells (Figure 6A). 

As determined by a 3-(4,5-dimethyl-2-thiazolyl)-2,5-diphenyltetrazolium bromide (MTT) assay, Mito-CP in 0.01–10 μM dose ranges effectively decreased cell viability of C643, TPC-1, and 8505C cells within 48 h, with all cell lines exhibiting very high sensitivity to this drug (Figure 6B,C); IC_50_ values were calculated at 0.43 μM for C643, 0.78 μM for TPC-1, and 0.33 μM for 8505C. Mito-CP consists of the CP moiety, a 5-membered nitroxide that has antioxidant properties, and the TPP moiety that mediates mitochondria targeting – the chemical structure of Mito-CP is depicted in Figure S2 of ref [18]. In contrast to Mito-CP, equal doses of TPP did not significantly decrease cell viability of these cells, as determined with C643 and TPC-1 cells, indicating the specificity of Mito-CP (Figure 6B). We also assessed Mito-CP for its efficacy relative to the FDA-approved kinase inhibitors. As determined by MTT assay after 48 h drug treatments (Figure 6C), Mito-CP was about 10 times more potent than the B-Raf inhibitor PLX4032 in 8505C cells (IC_50_ values 0.33 μM vs 3.42 μM) and also far more potent than the tyrosine kinase inhibitors, vandetanib and cabozantinib, in TPC-1 cells (IC_50_ values 0.25 μM vs 2.12 μM and 8.92 μM). 

**Figure 7:**
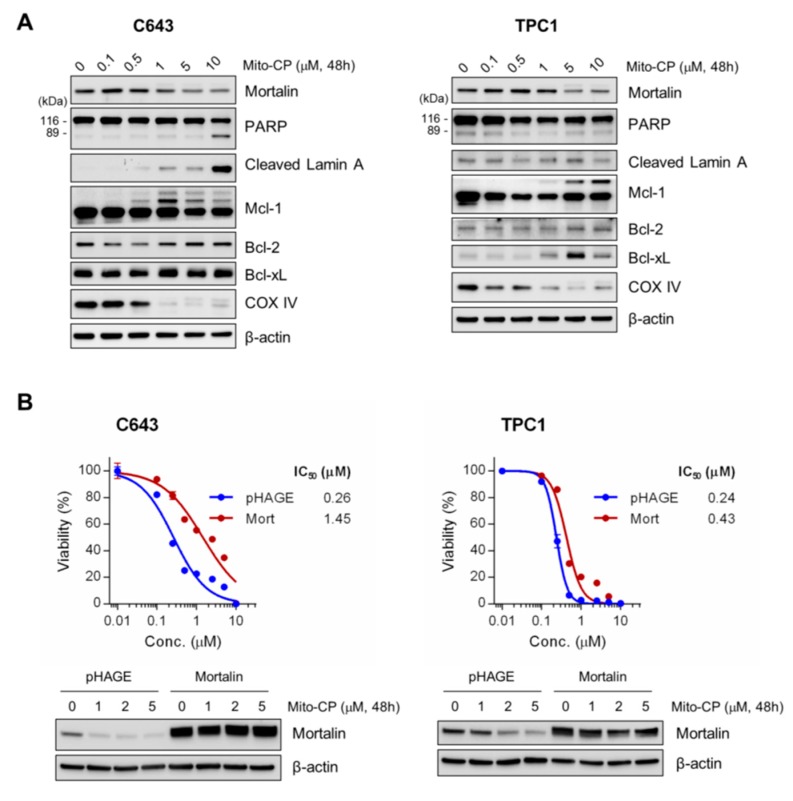
Mito-CP induces mortalin downregulation while mortalin overexpression confers PTC and ATC cells partial resistance to Mito-CP. (**A**) Total lysates of C643 and TPC-1 cells treated with different doses of Mito-CP for 48 h were analyzed by Western blotting for expression of the indicated proteins. β-actin is the control for equal amounts of protein loading. (**B**) C643 and TPC-1 cells infected with lentiviral pHAGE expressing mortalin were treated with serially increasing doses of Mito-CP for 48 h. Cells were then allowed to recover in a drug-free fresh medium for 24 h prior to measuring cell viability by an MTT assay. Data (mean ± SD, *n* = 4) are expressed as the percentage of untreated control. Western blotting of total cell lysates indicates the degree of mortalin overexpression.

To determine the nature of Mito-CP-mediated suppression of these tumor cells, we conducted Western blot analyses of total lysates of C643 and TPC-1 cells treated with Mito-CP for 48 h. Consistent with its mitochondria damaging effects, Mito-CP decreased COX IV levels in both cell lines in a dose-dependent manner (Figure 6A). Under these conditions, we found that Mito-CP robustly induced the cleavage of lamin A and PARP in C643 cells but not in TPC-1 cells, while increasing the levels of Bcl-xL in TPC1 cells (Figure 7A). Mito-CP also significantly altered the pattern of Mcl-1 expression in both cell lines although it did not affect the levels of another anti-apoptotic Bcl-2 family member, Bcl-2, in these cells (Figure 7A). Intriguingly, Mito-CP downregulated mortalin levels in a dose-dependent manner in both C643 and TPC-1 cells (Figure 7A) while the lentiviral vector-mediated mortalin overexpression conferred partial resistance to Mito-CP (Figure 7B), suggesting that mortalin downregulation may be a mechanism underlying the toxicity of Mito-CP. These data suggest that Mito-CP can effectively suppress not only MTC but also PTC and ATC cells.

## 3. Discussion

Targeted therapy using the small molecule inhibitors of oncogenic receptor tyrosine kinases or molecular switches has been significantly advanced in recent years for the treatment of surgically incurable progressive thyroid cancers, e.g., RET and VEGF inhibitors for MTC, and B-Raf and MEK1/2 inhibitors for B-Raf^V600E^ PTC and ATC. Nevertheless, these therapies have limits because not all patients are responsive while tumors usually progress after initial response [4,5]. Therefore, expanding the repertoires of potential therapeutic targets is demanded. Our previous studies [13,15] and current data demonstrate that mortalin is necessary for tumor cell proliferation and survival in different types of thyroid tumors.

Our analyses of patient tumor biopsies and cell lines demonstrate that mortalin is upregulated not only in MTC but also in PTC, FTC and ATC, broadening the potential significance of mortalin in additional thyroid tumor types beyond MTC. Moreover, our previous studies in MTC cells [13] and current analysis of C643 ATC cells consistently detected mortalin mainly in mitochondria, which supports our hypothesis that mitochondrial mortalin is critical for thyroid tumor cell proliferation and survival by virtue of its role in regulating mitochondrial bioenergetics and redox balance. Noteworthy is that mortalin is also detected in different subcellular compartments, especially in cancer cells, suggesting its functional diversity [8,9,10]. Indeed, mortalin has been shown for its ability to sequester TP53 in the cytosol, which leads to inactivation of the tumor suppressor in different tumor types [19,20,21,22,23]. Mortalin also affected Ras activity through its interaction with mevalonate pyrophosphate decarboxylase, an enzyme that biochemically modifies Ras [24]. Although the exact location for the regulation is yet unclear, we have recently demonstrated that mortalin negatively regulates the activity of the Raf/MEK/ERK pathway by promoting the physical interaction between protein phosphatase 1 alpha and MEK1/2 [25]. Of note, this ability of mortalin to modulate the Raf/MEK/ERK pathway was important for B-Raf^V600E^ melanoma and K-Ras^G12V^ colon carcinoma cells to bypass the cytostatic effects associated with high MEK/ERK activity [12]. This observation has not been extended to B-Raf^V600E^ thyroid cancer cells, requiring a future study. Given these diverse roles of mortalin, it may be possible that different types of tumor cells may require different aspects of mortalin functions. This may partly account for the differential mortalin knockdown effects on the cell cycle regulators in PTC and ATC cell lines. In support of this notion, pathophysiological characteristics of thyroid cancer are also determined by a specific genetic background, e.g., the mutational status of *TP53* or *hTERT* [26,27,28]. Of note, while we previously demonstrated that MKT-077, a mortalin inhibitor, can suppress MTC xenografts in mice without causing serious toxicity [14], there has been effort to improve the efficacy and bioavailability of this compound [29]. As such, it will be important to evaluate advanced MKT-077 analogs in different thyroid tumor models.

Mitochondria are often dysregulated in cancer, and increased tumor cell dependency on mitochondria provides an opportunity to design a novel therapeutic strategy [30]. We previously demonstrated therapeutic potential of mitochondria targeting for MTC [13,14,15], and the present study suggests that the potential of mitochondria targeting may be extended to a broader spectrum of thyroid tumor types. As such, additional studies are required to evaluate various strategies that utilize mitochondria-targeted agents in thyroid cancer. For example, tumor suppressive effects of TPP-conjugated metabolism interfering agents are attributed to their Δψ_m_–dependent enrichment in mitochondria, and subsequent disruption of energy metabolism and redox balance in tumor cells [31,32]. We demonstrated that, when combined with vandetanib and cabozantinib, TPP-based compounds more effectively suppressed MTC cells because these tyrosine kinase inhibitors augmented Δψ_m_–dependent uptake/retention of TPP-conjugated agents in MTC cells. It is important to determine whether similar effects could be induced in different thyroid tumor types for which tyrosine kinase inhibitors are considered for therapy. Importantly, vandetanib and cabozantinib potentiated Mito-CP not only in drug naïve but also in drug-resistant MTC cells, suggesting the potential benefit of this strategy for drug-resistant MTC. In support, Mito-CP was also effective in suppressing PLX4032-resistant B-Raf^V600E^ melanoma cells [18], demanding a more thorough evaluation in B-Raf^V600E^ thyroid tumors. 

In summary, this study demonstrates that mortalin is upregulated not only in MTC but also in PTC, FTC, and ATC and that mortalin is also necessary for tumor cell proliferation and survival in these tumor types. Moreover, this study demonstrates that Mito-CP can effectively suppress PTC and ATC cells that express RET/PTC or B-Raf^V600E^. Combined with our previous findings, this report expands the significance of mortalin and the therapeutic potential of mitochondria-targeting strategies to a broader spectrum of thyroid cancer.

## 4. Materials and Methods

### 4.1. Cell Culture and Reagents

TPC-1 (ATCC, Manassas, VA) and 8505C (ATCC) were maintained in RPMI 1640 (Invitrogen, Carlsbad, CA, USA) supplemented with 10% fetal bovine serum, 100 U of penicillin and 100 μg of streptomycin per ml. C643 (ATCC) was maintained in the same medium additionally supplemented with 292 mg/L glutamine. FTC133 was obtained from B. Nelkin (Johns Hopkins Medical Institute) and maintained in DMEM/Ham’s F-12 medium (Invitrogen). These media were supplemented with serum and antibiotics as above. The progenies of PTC-1 pTRIPZ-dox-shMort and C643 pTRIPZ-dox-shMort were generated by clonal selection after prolonged cell culture in the presence of puromycin. 

Mito-CP [33] was obtained from B. Kalyanaraman (Medical College of Wisconsin). Methyl-triphenyl-phosphonium (TPP), puromycin, G418, and doxycycline were purchased from Sigma-Aldrich (St Louis, MO, USA). Vandetanib was purchased from LC Laboratories (Woburn, MA, USA). PLX4032 and cabozantinib were purchased from Selleck Chemicals (Houston, TX, USA). Chemical structures of Mito-CP and TPP are depicted in Figure S2 of ref [18].

### 4.2. RNA Interference and Recombinant Lentiviral Constructs

Construction of the pLL3.7 lentiviral mortalin-targeting shRNA constructs (shMort #1 and shMort#2) was previously described [12]. The *pTRIPZ* doxycycline-inducible microRNA-adapted shRNA targeting human mortalin was purchased from Open Biosystems (V3THS_362249). pHAGE-HA-Mort* was previously described [12]. Lentivirus was produced as previously described [12].

### 4.3. Cell Proliferation, Death, and Cell Cycle Assays

For the assay using MTT, cells were incubated with culture medium containing MTT (0.5 mg/mL, Sigma) in 96 well-plates for 2 h at 37 °C, switched into 200 μL of dimethyl-sulfoxide, and shaken for 5 min at room temperature before measuring absorbance at 540 nm, as previously described [12]. Cell viability was also measured by counting trypan blue-stained cells using hemocytometer. Annexin V (Invitrogen) was stained according to manufacturer’s instruction. Cell cycle analysis was conducted using Hoechst 33342 as previously described [15].

### 4.4. Immunoblot Analysis 

Immunoblotting was performed as previously described [34]. Antibodies were diluted as follows: mortalin (sc-13967), 1:2500; glyceraldehyde-3-phosphate dehydrogenase (GAPDH), 1:5000; RET, 1:1000; p21^CIP1^, 1:2500; p27^KIP1^, 1:2000; TP53, 1:1000; E2F-1, 1, 1000; anti-HA, 1:1000 (Santa Cruz Biotechnology, Santa Cruz, CA); poly(ADP-ribose) polymerase (PARP), 1:1000; cleaved lamin A, 1:2000; Bcl-2, 1:2000; Bcl-xL, 1:2000; Mcl-1, 1:2000; β-actin, 1:10,000; COX IV, 1:2000 (Cell Signaling Technology, Danvers, MA); p16^INK4A^, 1:2500 (BD Bioscience, San Jose, CA). Images of immunoblots were taken and processed using ChemiDoc XRS+ and Image Lab 3.0 (Bio-Rad, Hercules, CA).

### 4.5. Immunofluorescence 

Cells grown on chamber slides were washed with PBS, fixed with 3% paraformaldehyde, and incubated overnight at 4 °C with the monoclonal antibody specific to mortalin (1:200, sc-133137, Santa Cruz Biotechnology) or the polyclonal antibody specific to COX IV (1:300, #4850, Cell Signaling Technology). Cells were then double-stained with secondary anti-mouse Alexa Fluor 594 and anti-rabbit Alexa Fluor 488 (Invitrogen) conjugates at 1:200 dilution as described previously [35]. Images were acquired using a LCM510 microscope (Zeiss) and analyzed with proprietary AIM 4.2 software.

### 4.6. Immunohistochemistry

Formalin-fixed, paraffin-embedded 5 µm sections of non-overlapping thyroid carcinoma tissue microarrays (TH208, TH801, TH802, and TH806), containing 71 PTC cases, 39 FTC cases, 12 ATC cases, 39 benign thyroid tumor cases, and 55 normal thyroid samples, were purchased from US Biomax (Rockville, MD). The manufacturer-supplied de-identified clinical information is listed in Supplementary Materials, Table SI. All tissue samples consisted of uniform cores, 1.5 mm in diameter. The specimens were analyzed using monoclonal anti-mortalin antibody, D-9 (Santa Cruz Biotechnology, sc-133137), as previously described [12]. The specificity of this staining was validated with normal IgG type 2.

### 4.7. Statistical Analysis 

Unless otherwise specified, a two-tailed unpaired Student’s t-test was used to assess the statistical significance of two data sets. The significance of immunohistochemistry data of human MTC tissues was determined by the Kruskal–Wallis test (nonparametric ANOVA) with Dunn post-test for multiple comparisons. *p* values of < 0.05 were considered statistically significant.

## Supplementary Materials

Supplementary materials can be found at https://www.mdpi.com/1422-0067/20/9/2069/s1.
