# Supplementary figures and images for: Mortalin (GRP75/HSPA9) Promotes Survival and Proliferation of Thyroid Carcinoma Cells
Source: Int J Mol Sci. 2019 Apr 26;20(9):2069. doi: 10.3390/ijms20092069 (PMC6540051; doi:10.3390/ijms20092069)

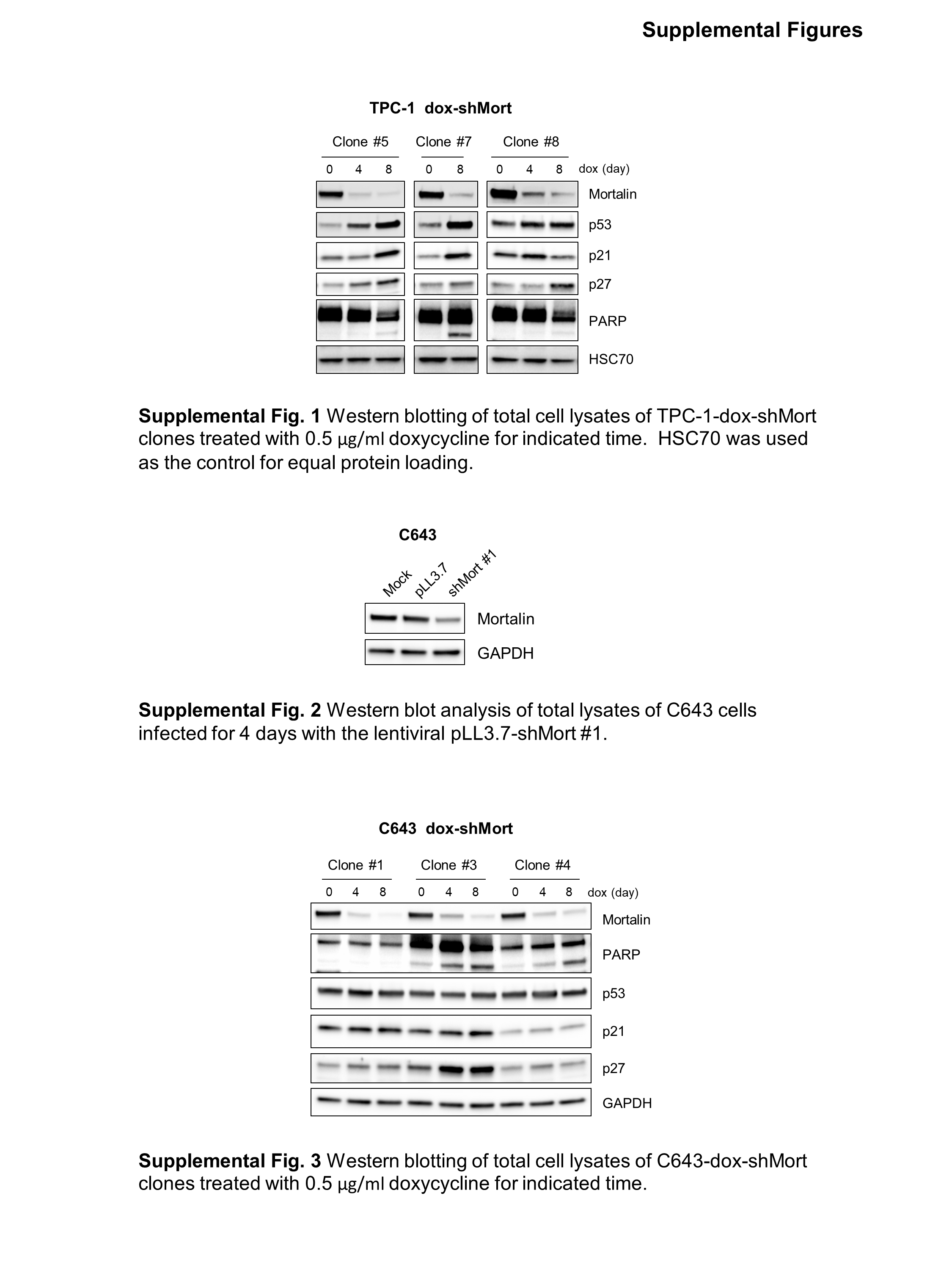

Supplement: Supplementary file 1 [file ijms-20-02069-s001.zip › Supplemental figures.TIF]
